# DspA/E Contributes to Apoplastic Accumulation of ROS in Non-host *A. thaliana*

**DOI:** 10.3389/fpls.2016.00545

**Published:** 2016-04-26

**Authors:** Alban Launay, Oriane Patrit, Estelle Wénès, Mathilde Fagard

**Affiliations:** ^1^CNRS 3559, Institut Jean-Pierre Bourgin, INRA, AgroParisTech, ERL, Université Paris-SaclayVersailles, France; ^2^Université Paris-Sud–Université Paris-SaclayOrsay, France; ^3^AgroParisTechParis, France

**Keywords:** non-host, *Erwinia amylovora*, *Arabidopsis thaliana*, ROS, DspA/E

## Abstract

The bacterium *Erwinia amylovora* is responsible for the fire blight disease of *Maleae*, which provokes necrotic symptoms on aerial parts. The pathogenicity of this bacterium in hosts relies on its type three-secretion system (T3SS), a molecular syringe that allows the bacterium to inject effectors into the plant cell. *E. amylovora-*triggered disease in host plants is associated with the T3SS-dependent production of reactive oxygen species (ROS), although ROS are generally associated with resistance in other pathosystems. We showed previously that *E. amylovora* can multiply transiently in the non-host plant *Arabidopsis thaliana* and that a T3SS-dependent production of intracellular ROS occurs during this interaction. In the present work we characterize the localization and source of hydrogen peroxide accumulation following *E. amylovora* infection. Transmission electron microscope (TEM) analysis of infected tissues showed that hydrogen peroxide accumulation occurs in the cytosol, plastids, peroxisomes, and mitochondria as well as in the apoplast. Furthermore, TEM analysis showed that an *E. amylovora dspA/E-*deficient strain does not induce hydrogen peroxide accumulation in the apoplast. Consistently, a transgenic line expressing DspA/E accumulated ROS in the apoplast. The NADPH oxidase-deficient *rbohD* mutant showed a very strong reduction in hydrogen peroxide accumulation in response to *E. amylovora* inoculation. However, we did not find an increase in bacterial titers of *E. amylovora* in the *rbohD* mutant and the *rbohD* mutation did not suppress the toxicity of DspA/E when introgressed into a DspA/E-expressing transgenic line. Co-inoculation of *E. amylovora* with cycloheximide (CHX), which we found previously to suppress callose deposition and allow strong multiplication of *E. amylovora* in *A. thaliana* leaves, led to a strong reduction of apoplastic ROS accumulation but did not affect intracellular ROS. Our data strongly suggest that apoplastic ROS accumulation is one layer of the non-host defense response triggered by the type three effector (T3E) DspA/E, together with callose deposition.

## Introduction

One of the most common and earliest responses of plants to pathogens is reactive oxygen species (ROS) production (Lamb and Dixon, 1997). In the case of biotrophic pathogens, ROS production is usually associated with defense (Torres et al., 2002) but in the case of necrotrophic pathogens the role of ROS production is less clear. For example, the necrotrophic fungus *Botrytis cinerea* induces the production of hydrogen peroxide (H_2_O_2_) that is associated with disease (van Baarlen et al., 2007). It was also shown that the NbrbohB-dependent production of H_2_O_2_ following infection by *B. cinerea* contributes to lesion development in *Nicotiana benthamiana* (Asai and Yoshioka, 2009). In the case of the necrotrophic bacterium *Dickeya dadantii*, strong oxidative stress was observed in *Arabidopsis thaliana* and it was shown that this accumulation contributes to defense (Fagard et al., 2007). In *A. thaliana*, RbohD was found to be the main source of ROS production to the avirulent strain of *Pseudomonas syringae* avrRpm1 (Torres et al., 2002) and to the bacterial necrotroph *D. dadantii* (Fagard et al., 2007). The current knowledge on RbohD, which encodes an NADPH oxidase, has been recently reviewed (Marino et al., 2012).

Non-host resistance is a widespread process in plants that corresponds to the resistance of a plant species to all the isolates of a given pathogen species that is pathogenic on other plant species (Schulze-Lefert and Panstruga, 2011). Indeed, most plants are resistant to most pathogens and disease remains the exception. While non-host resistance of plants to pathogenic fungi has attracted some attention, only a few studies have focused on non-host resistance of plants to bacterial pathogens. Several examples of the role of ROS accumulation during non-host interactions have been described in the literature. For example, Senthil-Kumar and Mysore showed in 2012 that *A. thaliana* deficient mutants *δoat* and *p5cdh*, which are deficient for ROS accumulation, are compromised in the non-host resistance to *P. syringae* pv. *tabaci*. Likewise, suppression of glycolate oxidase genes compromised H_2_O_2_ accumulation in *A. thaliana* in response to *P. syringae* pv. *tabaci* which is associated with more bacterial multiplication (Rojas et al., 2012). Another example is the non-host interaction between *Pinus pinaster and B. cinerea* in which a biphasic ROS accumulation is associated to a hypersensitive response (Azevedo et al., 2008).

*Erwinia amylovora* is the causal agent of bacterial fire blight, which affects rosaceae plants mainly of the pyreae tribe. The pathogenicity of *E. amylovora* depends mainly on the type three secretion system (T3SS) which secretes and injects type three effectors (T3Es) inside the plant cell; among them, the T3E DspA/E plays a major role as a *dspA/E* deficient mutant is non-pathogenic (Barny et al., 1990). In host plants, *E. amylovora* induces ROS accumulation and induces the activity of ROS detoxifying enzymes such as glutathion-*S*-transferases (Venisse et al., 2003). ROS accumulation in host leaves is T3SS-dependent as a non-pathogenic type three secretion mutant does not induce ROS accumulation (Venisse et al., 2001). *E. amylovora* was found to be approximately 10 times less susceptible to oxidative stress *in vitro* than the biotrophic bacterium *P. syringae* pv. *tomato*, leading the authors to hypothesize that oxidative stress induced by *E. amylovora* was required for cell death induction and disease (Venisse et al., 2003).

We showed previously that in non-host *A. thaliana*, *E. amylovora* is able to multiply transiently and induce necrotic symptoms but that *A. thaliana* mounts an efficient defense, which leads to a decrease in bacterial titers after 48 h (Barny et al., 2008; Degrave et al., 2008; Moreau et al., 2012). Multiplication of *E. amylovora* in *A. thaliana* leaves is dependent on the DspA/E effector protein and associated with the production of necrotic symptoms, as in host plants. Indeed, transgenic plants expressing DspA/E, allowed a *dspA/E*-deficient mutant to multiply *in planta* (Degrave et al., 2013). Although, non-host resistance of *A. thaliana* toward *E. amylovora* has not been elucidated, our previous work showed that it requires an activation of plant defense since co-inoculation with cycloheximide (CHX), a repressor of protein synthesis, led to an important multiplication of *E. amylovora* in *A. thaliana* (Moreau et al., 2012).

In the present work, we analyze in detail ROS production during the non-host interaction between *E. amylovora* and *A. thaliana* and we analyze the role of DspA/E in this ROS production. Our results demonstrate that *E. amylovora* triggers DspA/E-independent intracellular H_2_O_2_ accumulation and DspA/E-dependent apoplast accumulation of H_2_O_2_. We show that RbohD is involved in H_2_O_2_ accumulation and suppression of necrosis but we did not find any evidence that it was required for resistance. Co-inoculation with cycloheximide (CHX), previously shown to allow a strong increase in bacterial titers, did not affect intracellular ROS accumulation but strongly reduced apoplastic ROS accumulation. Altogether, our results suggest a role for apoplastic H_2_O_2_ accumulation in non-host resistance.

## Materials and Methods

### Plant Lines and Bacterial Strains Used in This Study

Seeds of *A. thaliana* Col-0 were obtained from the INRA Versailles collection. Plants were grown for 5 weeks in soil and were subjected to an 8 h-light and 16 h-dark cycle at 21°C (day)/18°C (night) with 65% relative humidity. Seeds of the *rbohD* mutant (Torres et al., 2002) were obtained from J. Dangl (Chapel Hill, NC, USA). The double *rbohD*/13-1-2 line was obtained in the laboratory by crossing.

*Erwinia amylovora* wild-type strain CFBP1430 (*Ea*), T3SS-deficient strain CFBP6023 (*tts*) were obtained from the Collection Française des Bactéries Phytopathogènes (CFBP). The *E. amylovora dspA/E-*deficient strain (*dspA/E*) is described in Degrave et al. (2013). For plant inoculation, a liquid pre-culture of each strain was plated on LB and grown overnight at 28°C.

The 7-2-1 GFP-expressing *A. thaliana* transgenic line and the 13-1-2 *dspA*/*E*-expressing *A. thaliana* transgenic line are described in Degrave et al. (2013). For root growth measurement, estradiol treatment was performed on 6-days-old seedlings grown on 1/5th Murashige and Skoog supplemented with 1% sucrose. A stock solution of estradiol was initially prepared in Dimethyl sulfoxide (DMSO) at 20 mM and stored at −20°C. The root of each seedling was treated with a drop of 200 μL of an estradiol solution (diluted to the indicated concentration in water), or mock-treated for 10 min and transferred to a growth chamber (16 h day/8 h night). Pictures were taken 24 h after treatment and root length measurement was performed using the ImageJ software. For ROS analysis, a 5 μM estradiol solution was infiltrated into leaves of 5-weeks-old rosette that were sampled and stained at the indicated time point.

### Bacterial Infection

A bacterial suspension of *E. amylovora* with an OD of 0,1 (10^7^ CFU/mL) in water was syringe-inoculated into 5-weeks-old plants. For symptom intensity and bacterial count the procedure is described in Degrave et al. (2008). For CHX treatment, *E. amylovora* was co-inoculated with the translation inhibitor cycloheximide (Sigma) at 4 μg/ml as described in Moreau et al. (2012).

### Detection of ROS

2′,7′-Dichlorofluorescein diacetate (DCFH-DA) staining was used to detect intracellular hydrogen peroxide. A 30 mM DCFH-DA (Sigma) solution was prepared in DMSO and diluted 100 times in deionized water. Inoculated or mock-treated leaves were collected 16 h post-inoculation (hpi) and vacuum-infiltrated with DCFH-DA. Leaves were immediately put on a microscope slide and fluorescence emission was observed under a binocular magnifier with a GFP filter (510 nm).

Diaminobenzidine (DAB) staining was used to detect intra- and extracellular hydrogen peroxide (H_2_O_2_). A 5 mg/mL DAB (Sigma) stock solution pH 3,7 was prepared with deionized water and diluted to a final concentration of 1 mg/ml. Five-weeks-old leaves were collected 2 h after inoculation (as indicated) and vacuum infiltrated with the DAB solution. Leaves were then placed in a wet Petri dish overnight and the staining was stopped at 16 hpi by placing the leaves in ethanol to discolor them. Once all chlorophyll was removed, leaves were mounted in water between a microscopic slide and a cover glass and observed with a Nikon Microphot FXA photonic microscope at X400 magnification.

### Transmission Electron Microscopy (TEM) Observations

For the transmission electron microscopy (TEM) observations, pieces of 0,1 by 0,3 mm were sampled from mock-treated or inoculated leaves as described. Samples were incubated in 5 mM cerium chloride (CeCl_3_) in MOPS buffer 50 mM pH 7.2 during 1 h at room temperature. Samples were fixed in 0,1 M cacodylate buffer (1,5% paraformaldehyde and 2% glutaraldehyde pH 7,2) during 4 h under agitation with a 1 min vacuum infiltration every hour. Samples were then rinsed four times in cacodylate buffer during 15 min under agitation and placed in the contrast solution (cacodylate 0.1 M pH 7.2, Osmium Tetroxide) during 1 h. Samples were rinsed three times with deionized water during 10 min under agitation and subjected to increasing dehydration with ethanol 25, 50, 70, 85% during 30 min and finally in ethanol 90% overnight at 4°C. Samples were then subjected to absolute ethanol three times during 30 min. Samples were treated with 50% absolute ethanol/50% propylene oxide during 30 min under agitation followed by three 100% propylene oxide baths. Embedding consisted in an overnight bath of 2/3 propylene oxide, 1/3 Epon-araldite (Electron Microscopy Sciences) at room temperature and a second bath with 1/3 propylene oxide, 2/3 Epon-araldite applied for 9 h. Following propylene oxide evaporation, two baths with pure Epon-araldite resin were applied. Samples were placed in silicon flat molds and resin was polymerized at 60°C for 48 h. Semi-thick sections were made with a razor blade and a Histome diamond knife for quality control of each sample (stained with 0,1% toluidine blue in water). Finally, samples were cut with an ultra-thin section diamond knife (70 nm). Cut samples were collected on electron microscope grids (200 mesh) for further observation on a Zeiss EM912 OMEGA electron microscope at a magnification of 10000 to 50000 X.

### Statistical Analysis and ImageJ Software

The Mann and Whitney test was selected because we compared quantitative values with two independent conditions with less than 30 samples per condition. Intensity of DAB staining or DCFH-DA fluorescence was quantified using the ImageJ software (version 1.46r). The intensity was measured in a square section of the half-blade that was inoculated using the “Integrated density” (intdens) function of the software. This operation was repeated on 5–10 leaves per condition depending on the experiment. The results presented correspond to the mean intensity for each condition for a given experiment. All experiments were performed three times and a representative experiment is showed. The significant differences presented in the figures were found in each independent experiment.

## Results

### *Erwinia amylovora* Induces Accumulation of Intracellular and Extracellular Hydrogen Peroxide

**FIGURE 1:**
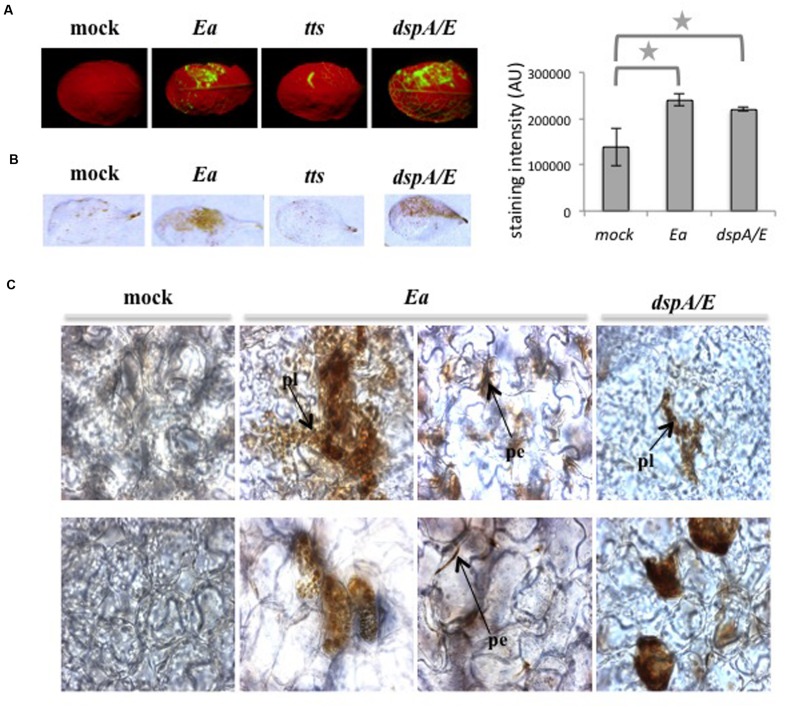
***Erwinia amylovora* induces accumulation of H_2_O_2_.** **(A–C)** Leaves of 5-weeks-old plants were infiltrated on the upper half of the leaf blade with water (mock), *E. amylovora* (*Ea*) wild-type strain, T3SS-deficient mutant (*tts*) or *dspA/E* mutant (*dspA/E*). **(A)** Leaves stained with DCFH-DA show intracellular H_2_O_2_ accumulation in green. **(B)** Brown stains indicate accumulation of H_2_O_2_ in leaves stained with DAB, the graph to the right shows DAB staining intensity. The stars indicate a significant difference according to Mann and Whitney’s statistical test (*p* < 0.05). **(C)** Localization of H_2_O_2_ deposits in DAB-stained leaves observed under a photon microscope. Bar: 50 μm; pl: plastid; pe: staining at the periphery of the cell. Similar results were obtained for three independent experiments. Top panel: palissadic parenchyma; bottom panel: spongy parenchyma.

We showed previously that *E. amylovora* induces the accumulation of intracellular ROS detected by the fluorescent probe DCFH-DA (Degrave et al., 2008; **Figure 1A**), which allows detection of hydrogen peroxide. In order to determine the localization of H_2_O_2_ accumulation in response to *E. amylovora* inoculation, we used diaminobenzidine (DAB), which forms a brown precipitate in the presence of H_2_O_2_. Five-weeks-old *A. thaliana* rosette leaves were infiltrated with *E. amylovora* and the accumulation of H_2_O_2_ was determined at 16 hpi. Most *E. amylovora-*infected leaves (84%) displayed strong DAB staining (**Figure 1B**). In general, mock-inoculated leaves displayed no DAB staining but a small proportion of leaves (18%) displayed slight DAB staining (**Figure 1B**). We then observed the DAB-stained leaves by photon microscopy, which revealed that mock-infected leaves displayed only very slight diffuse brown staining while wild-type *E. amylovora*-infected leaves displayed distinct brown staining in groups of cells in both the palisade and the spongy parenchyma (**Figure 1C**). The brown precipitate accumulated both inside the cells and at their periphery. In the groups of stained cells, numerous round organelles were distinctly stained with brown precipitate (**Figure 1C**), the number and size of which suggest that they could be plastids.

Altogether, our results confirm that infection by *E. amylovora* of the non-host plant *A. thaliana* leads to hydrogen peroxide accumulation in numerous round organelles as well as at the periphery of cells, however due to the weak resolution of the light microscope technique, the precise localization could not be determined.

### A *dspA/E*-Defective *E. amylovora* Mutant Can Induce ROS Accumulation in *A. thaliana*

Our previous work shows that during the interaction between *A. thaliana* and *E. amylovora*, the T3SS is responsible for intracellular ROS accumulation detected by DCFH-DA (Degrave et al., 2008; **Figure 1A**). Among the known effectors to be injected by *E. amylovora* inside plant cells, DspA/E plays a crucial role during disease-related ROS accumulation on host plants (Venisse et al., 2003) and during the interaction with *A. thaliana* (Degrave et al., 2013). In order to determine the role of DspA/E in ROS production, we analyzed ROS accumulation following inoculation with a *dspA/E*-defective mutant using DCFH-DA and DAB staining. We also used a T3SS-defective strain for comparison. Consistent with our previous results, the T3SS-defective strain did not induce ROS accumulation detected by DCFH-DA (**Figure 1A**). Consistently, leaves inoculated with a T3SS-defective strain (**Figure 1B**) did not accumulate ROS detected by DAB staining.

In contrast, our results show that leaves inoculated with a *dspA/E*-defective mutant accumulate both DCFH-DA and DAB-detectable ROS (**Figures 1A,B**). At the tissue level, leaves inoculated with a *dspA/E-*defective mutant accumulated ROS in both palisade and spongy parenchyma and intracellular staining could be observed, in particular in the round organelles (**Figure 1C**).

Altogether, our results show that a *dspA/E*-deficient strain, contrary to a T3SS-deficient strain, remains able to trigger H_2_O_2_ accumulation in *A. thaliana*, indicating that other elements of the T3SS than DspA/E are involved in ROS accumulation during non-host resistance.

### *E. amylovora* Induces Strong H_2_O_2_ Accumulation in the Apoplast

**FIGURE 2:**
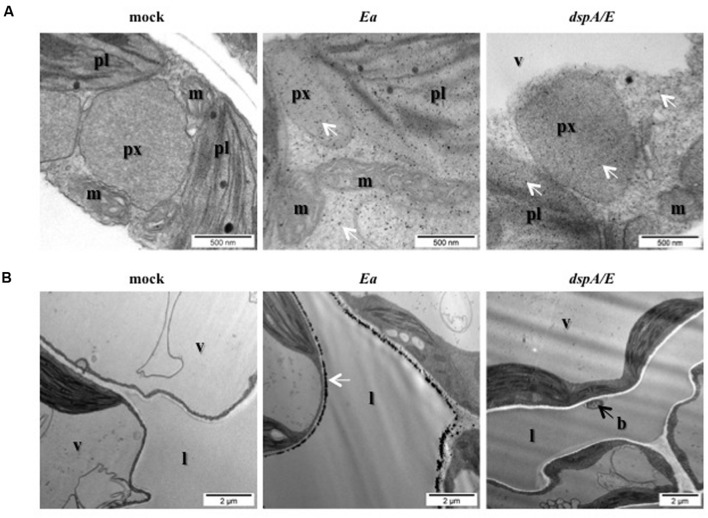
***Erwinia amylovora* induces strong H_2_O_2_ accumulation in the cytoplasm and in the apoplast.** **(A,B)** TEM observation of CeCl_3_-stained leaves. Black spots indicate H_2_O_2_ accumulation stained by CeCl_3_ (white arrows). Leaves of 5-weeks-old plants were infiltrated with water (mock), wild-type *E. amylovora* (*Ea*), or with the *dspA/E* mutant (*dspA/E*) and collected 16 hpi. **(A)** Bar: 500 nm. **(B)** Bar: 2 μm. b: bacterium, l: lacuna, m: mitochondrion, pl: plastid, px: peroxisome, v: vacuole.

In order to determine more precisely the localization of hydrogen peroxide accumulation, we decided to use CeCl_3_ staining which forms electron-dense deposits in the presence of hydrogen peroxide. Five-weeks-old *A. thaliana* plants were mock-treated or *E. amylovora*-inoculated, sampled 24 hpi and ultra-thin sections of CeCl_3_-stained leaves were observed with an electron microscope (**Figure 2**). The staining revealed that in *E. amylovora*-infected leaves hydrogen peroxide could be detected in all visible organelles: mitochondria, peroxisomes, chloroplasts as well as in the cytoplasm (**Figure 2A**). In addition, a very strong electron-dense staining was observed at the periphery of the cells, most likely in the apoplast, which was not observed in mock-inoculated leaves. Strong electron-dense staining was also observed around the bacteria, which could be seen in the apoplastic space or attached to the cell wall (**Figure 2B**).

Our results show that *E. amylovora* infection of non-host *A. thaliana* elicits the accumulation of hydrogen peroxide in different cellular compartments including chloroplasts, and reveals that *E. amylovora* triggers a strong hydrogen peroxide accumulation in the apoplast.

### DspA/E is Necessary for H_2_O_2_ Accumulation in the Apoplast

In order to determine the role of the T3E DspA/E in the triggering of ROS localized in the apoplast, we observed with TEM ultra-thin sections of CeCl_3_-stained leaves inoculated with the *dspA/E*-deficient strain. Our results indicate that intracellular ROS accumulated as in response to the wild-type strain (**Figure 2A**), which is consistent with our observations using DAB and DCFH-DA staining (**Figures 1A–C**). On the other hand, the strong ROS accumulation observed in the apoplast in leaves inoculated with the wild-type strain of *E. amylovora* was not observed in leaves inoculated with the *dspA/E*-deficient strain (**Figure 2B**).

**FIGURE 3:**
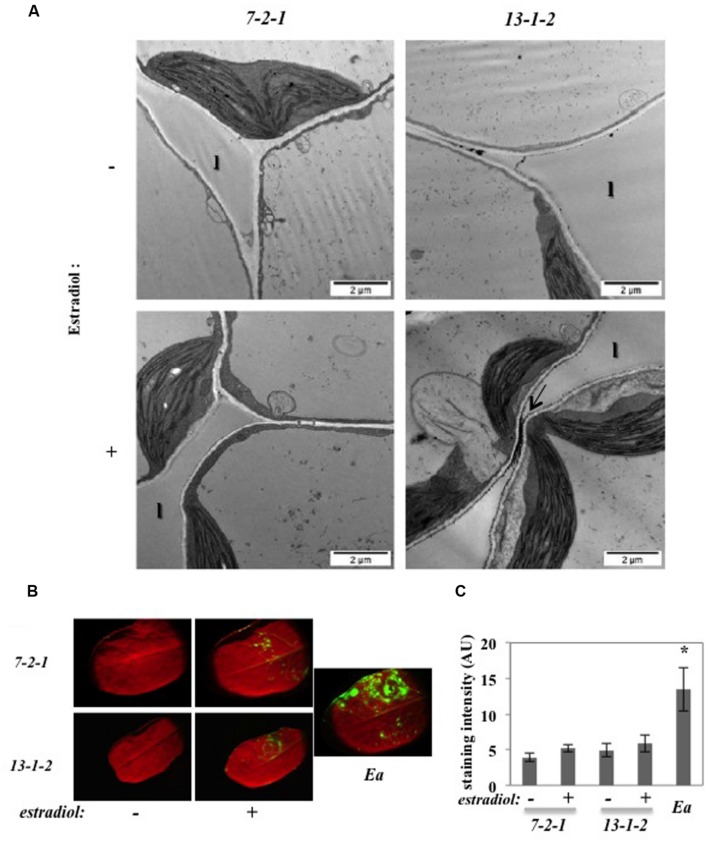
**DspA/E expressed *in planta* induces H_2_O_2_ accumulation in the apoplast.** **(A,B)** Leaves of 5-weeks-old plants were infiltrated with water (-) or a 5 μM estradiol solution (+) and collected 16 hpi. **(A)** TEM observation of CeCl_3_-stained leaves. Black spots indicate H_2_O_2_ accumulation stained by CeCl_3_ (arrow). Bar: 2 μm. l: lacuna. **(B)** DCFH-DA staining shows intracellular H_2_O_2_ accumulation in green. *E. amylovora (Ea)* inoculation was used as a positive control for DCFH-DA staining. **(C)** Mean DCFH-DA staining intensity, which was determined in 5–10 leaves per condition using the ImageJ Software. The asterisks indicate a significant difference according to Mann and Whitney’s statistical test (*p* < 0.05).

To confirm the role of DspA/E in the triggering of ROS in the apoplast, we used the 13-1-2 transgenic line expressing DspA/E under the control of an estradiol-inducible promoter that we described previously (Degrave et al., 2013). Ultrathin sections of CeCl_3_-stained leaves were observed by TEM (**Figure 3A**). Control plants (7-2-1) did not show any staining whether treated with estradiol or not (**Figure 3A**). In contrast, plants from the 13-1-2 line, bearing the bacterial *DspA/E* gene, treated with estradiol to induce *DspA/E* expression displayed staining in portions of the apoplast but no staining in the cytoplasm (**Figure 3A**). Mock-treated 13-1-2 plants also displayed slight staining in the apoplast which is consistent with low expression of *DspA/E* in mock-treated 13-1-2 plants as we showed previously (Degrave et al., 2013).

In order to confirm the specific role of DspA/E in triggering only apoplastic ROS, we stained the transgenic lines described above with DCFH-DA. As expected, the 7-2-1 control line did not show significant fluorescence while leaves inoculated with *E. amylovora*, used as a positive control, were clearly fluorescent (**Figure 3B**). The 13-1-2 line did not show significant fluorescence whether *DspA/E* expression was induced with estradiol or not (**Figure 3B**). The intensity of fluorescence was quantified using the ImageJ software (**Figure 3C**) and statistical analysis confirms that the level of fluorescence in the 13-1-2 line was the same as the control line whereas *E. amylovora*-inoculated leaves showed significant levels of fluorescence according to Mann and Whitney’s test (*p* < 0.05). These results confirm that DspA/E, when expressed in the plant cell, does not trigger significant intracellular H_2_O_2_ accumulation.

Our results clearly show that DspA/E is required and sufficient for accumulation of CeCl_3_-detectable ROS in the apoplast but does not trigger significant intracellular ROS.

### *rbohD* is Required for Intracellular H_2_O_2_ Accumulation and Limits Spread of Lesions

**FIGURE 4:**
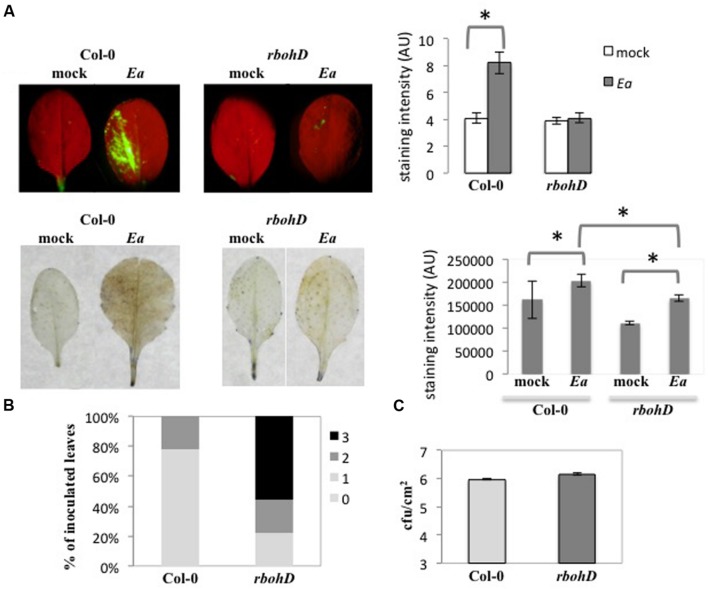
***rbohD* is required for intracellular H_2_O_2_ accumulation and limits spread of lesions.** **(A–C)** Leaves of 5-weeks-old plants were inoculated with wild-type *E. amylovora* (*Ea*) or mock-inoculated (mock) and stained to detect H_2_O_2_ accumulation 18 hpi. **(A)** Leaves stained with DCFH-DA show intracellular H_2_O_2_ accumulation in green (top), leaves stained with DAB show H_2_O_2_ accumulation in brown (bottom). The graphs on the right represent mean DCFH-DA intensity (top) and mean DAB staining intensity (bottom), which were determined in 5–10 leaves per condition using the ImageJ Software. The stars indicate a significant difference according to Mann and Whitney’s statistical test (*p* < 0.05). **(B)** Symptom intensity in Col-0 and *rbohD* mutant 24 hpi. We used a qualitative scale for the intensity of necrosis rating from no necrosis (0) to strong necrosis (3). Pictures corresponding to the scale can be found in Degrave et al. (2008). **(C)** Bacterial titers in Col-0 and *rbohD* mutant 24 hpi. No significant difference between titers in Col-0 and *rbohD* leaves according to Mann and Whitney’s statistical test (*p* < 0.05).

To determine the role of RbohD in the interaction between *A. thaliana* and *E. amylovora*, we measured ROS production in the *rbohD* knock-out (KO) mutant following *E. amylovora* inoculation (**Figure 4**). Using DCFH-DA, we did not detect significant fluorescence signal in the *rbohD* mutant inoculated with *E. amylovora* (**Figure 4A**) as we did in wild-type Col-0 plants (**Figures 1A** and **4A**). Using DAB staining, which detects H_2_O_2_ in both intra- and intercellular spaces, we found a weak brown staining in the *rbohD* mutant inoculated with *E. amylovora* (**Figure 4A**). The mock-treated leaves of both genotypes displayed no DAB- or DCFH-DA staining, as expected (**Figure 4A**). DCFH-DA staining intensity was not significantly higher in the *rbohD* mutant inoculated with *E. amylovora* compared to mock-treated *rbohD* plants (**Figure 4A**). DAB staining intensity was significantly higher in the *rbohD* mutant inoculated with *E. amylovora* compared to mock-treated *rbohD* plants but significantly lower than in wild-type inoculated plants (**Figure 4A**). These data indicate that RbohD is required for only part of the ROS accumulation that is triggered during the non-host resistance response of *A. thaliana* toward *E. amylovora*, and probably mostly intracellular ROS.

To understand the role of RbohD-dependent ROS accumulation during non-host resistance, we measured necrotic symptom intensity and bacterial titers following inoculation of *rbohD* mutant plants with *E. amylovora* (**Figure 4**). As described previously (Degrave et al., 2008), *E. amylovora* inoculation of wild-type Col-0 plants triggered necrotic symptoms that start to appear 24 hpi and can lead to strong necrosis after several days. In the *rbohD* mutant, the proportion of leaves with strong necrotic symptoms was significantly higher 24 hpi than in wild-type Col-0 plants (**Figure 4B**). This indicated that RbohD had a negative effect on necrosis development in leaves in response to *E. amylovora* inoculation. In contrast, we did not find any significant effect of the *rbohD* mutation on *E. amylovora* bacterial titers (**Figure 4C**), suggesting either that RbohD-dependent ROS production is not involved in non-host resistance against *E. amylovora* or that it is not the sole actor of non-host resistance.

**FIGURE 5:**
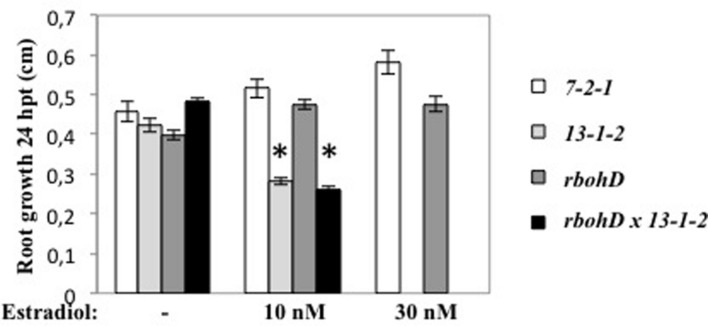
***rbohD* mutation does not suppress the toxicity of DspA/E.** Six-days-old seedlings were sown on MS/5 1% sucrose and treated or not (mock) with estradiol 10 or 30 nM. Root growth was measured 24 hpi. Asterisks represent significant differences according to Mann and Whitney’s test (*p* < 0.05).

DspA/E is known to induce cell death in both host and non-host plants. We showed previously that in the 13-1-2 line, induction of DspA/E expression with estradiol reduced seedling growth and ultimately led to the plant’s death (Degrave et al., 2013). To determine the effect of RbohD on this toxicity, we constructed a double homozygous line for the *rbohD* KO mutation and for the 13-1-2 transgene. Following estradiol treatment, the control line (7-2-1) and the *rbohD* mutant showed no reduction in root growth whereas the 13-1-2 line showed decreased root growth with increasing estradiol concentrations (**Figure 5**) as expected. Root growth of the double *rbohD* 13-1-2 line was reduced by estradiol in a similar manner as the 13-1-2 line (**Figure 5**). These data show that the *rbohD* mutation did not suppress DspA/E-induced cell death and toxicity in plant cells.

### Co-inoculation of *E. amylovora* with Cycloheximide (CHX) Reduces Apoplastic But Not Intracellular ROS Accumulation

**FIGURE 6:**
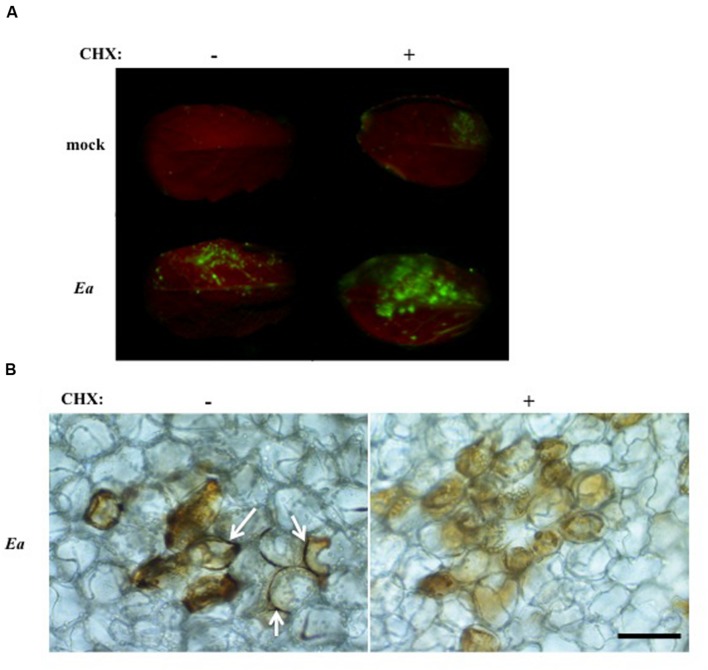
**Co-inoculation of *E. amylovora* with CHX reduces apoplastic ROS accumulation.** **(A,B)** Leaves of 5-weeks-old plants were mock-treated (mock) or inoculated with wild-type *E. amylovora* (*Ea*) and co-inoculated (+) or not (-) with 4 μg/ml cycloheximide. Leaves were stained with DCFH-DA or DAB to detect H_2_O_2_ accumulation 18 hpi. **(A)** Only the top half of the leaf blade was inoculated. Leaves stained with DCFH-DA show intracellular H_2_O_2_ accumulation in green. **(B)** Leaves stained with DAB show H_2_O_2_ accumulation in brown. Arrows indicate DAB staining in the apoplast. Bar: 50 μm.

We showed previously that co-inoculation of *A. thaliana* leaves with *E. amylovora* and CHX, an inhibitor of eukaryotic protein synthesis, led to a strong increase in bacterial titers compared to leaves inoculated with *E. amylovora* alone (Moreau et al., 2012). This led us to hypothesize that CHX co-inoculation suppressed the triggering of plant defense effective against *E. amylovora*. In order to determine the effect of CHX on intracellular and apoplastic ROS accumulation during the non-host response of *A. thaliana* to *E. amylovora*, we stained inoculated leaves with DCFH-DA or DAB as described earlier. In mock-inoculated leaves, no DCFH-DA fluorescence could be detected, whether the leaves were inoculated with water alone or with CHX (**Figure 6A**). In leaves inoculated with *E. amylovora*, DCFH-DA fluorescence was detected as described earlier but the staining was stronger in leaves co-inoculated with CHX (**Figure 6A**). These results indicated that CHX co-inoculation did not repress intracellular ROS accumulation triggered by *E. amylovora*.

To determine whether apoplastic ROS accumulation was affected by co-inoculation with CHX, we stained *E. amylovora*-inoculated leaves, co-inoculated with CHX or not, with DAB. The leaves were observed under a microscope to assess tissue localization of the staining as in **Figure 1C**. No DAB staining was observed in water or CHX-inoculated leaves (not shown). In leaves inoculated with *E. amylovora* alone, we found intracellular DAB staining as well as staining at the periphery of the cells as expected. In contrast, in leaves co-inoculated with *E. amylovora* and CHX we found only intracellular DAB staining and no staining at the periphery of the cells (**Figure 6B**). These results indicate that CHX represses the apoplastic ROS accumulation that is triggered by *E. amylovora* in *A. thaliana*.

## Discussion

*Erwinia amylovora* is a pathogenic bacterium with a narrow host range and the model plants *A. thaliana* and *N. benthamiana* are not considered as hosts for this bacterium. Indeed, in both cases the bacterium is able to multiply transiently in leaves after which bacterial multiplication is repressed by non-host resistance (Oh et al., 2007; Degrave et al., 2008). We showed previously that in *A. thaliana*, this non-host resistance is associated with the induction of several lines of defense including ROS accumulation (Moreau et al., 2012). ROS accumulation during plant pathogen interactions is generally associated with defense, in particular in the context of non-host resistance. In the case of *E. amylovora*, it has been suggested that ROS accumulation is critical for cell death activation and for disease, although this has not been formally demonstrated (Venisse et al., 2003). Thus, to better understand the role of ROS accumulation observed in *A. thaliana* leaves infected with *E. amylovora*, we investigated in detail the localization of this ROS accumulation and the role of the major T3E of *E. amylovora*, DspA/E, in this process.

Using a combination of ROS detection probes, we show in the present work that H_2_O_2_ accumulated both in the cytoplasm and in the apoplast in response to infection with *E. amylovora*. Moreover, TEM analysis showed that H_2_O_2_ accumulated in the cytosol and in several organelles, including mitochondria, peroxisomes and chloroplasts, as well as in the apoplast. Previous reports have shown similar localization of ROS accumulation during non-host interactions. For example, inoculation of lettuce with *P. syringae* pv. *phaseolicola* resulted in hydrogen peroxide accumulation in the apoplast (Bestwick et al., 1997) while inoculation of tobacco with *X. campestris* pv. *vesicatoria* led to hydrogen peroxide accumulation in the chloroplasts (Zurbriggen et al., 2009). Although, the specific role of the different sources of ROS in plant pathogen interactions is still not fully understood, ROS accumulation in mitochondria and chloroplasts are strongly linked to the triggering of cell death (Van Akent and Van Breusegem, 2015). Furthermore, it is now clear that ROS are more than deleterious molecules causing oxidative stress and that the spatiotemporal control of ROS production plays an important role in the specificity of ROS signaling (Mignolet-Spruyt et al., 2016). In particular, apoplastic ROS plays a role in both long-distance extracellular signaling and initiation of intracellular signaling.

Our data show that RbohD strongly contributes to H_2_O_2_ accumulation in response to *E. amylovora* inoculation in *A. thaliana*. The role of Rboh-dependent ROS in plant pathogen interactions is not clear as the corresponding *rboh* mutants show reduced, normal or increased cell death depending on the initial cell death trigger (Van Akent and Van Breusegem, 2015). However, we found that the *rbohD* mutation did not suppress *E. amylovora*-triggered necrosis or DspA/E toxicity. These results indicate that in *A. thaliana* DspA/E-triggered cell death does not require RbohD-dependent H_2_O_2_ accumulation. In contrast, we found that RbohD-dependent H_2_O_2_ accumulation was important for the restriction of necrosis since *rbohD* mutant plants showed necrotic symptoms of higher intensity than wild-type plants. Previous work indicated a role for RbohD in the limitation of spreading lesions (Torres et al., 2005), consistent with our observations.

**FIGURE 7:**
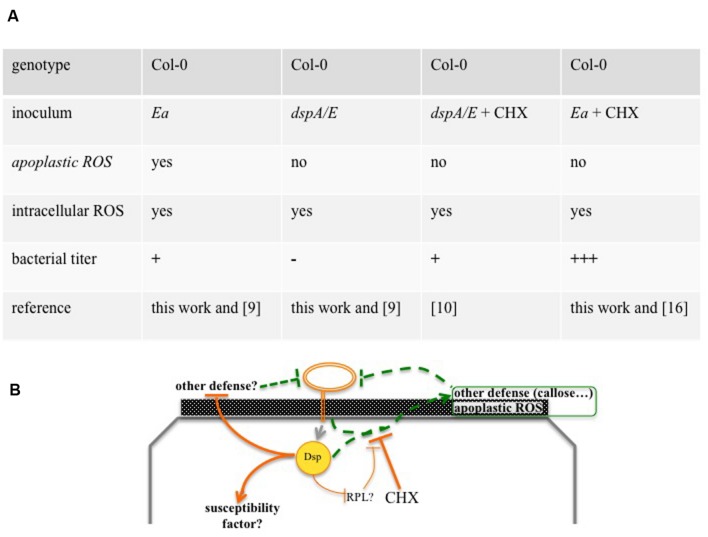
**Overview of *Arabidopsis thaliana* non-host resistance against *E. amylovora*.** **(A)** Overview of ROS production and bacterial growth in the different combinations of inoculum and genotype. Depending on the combination, ROS accumulate in the apoplast and/or in the cytoplasm or don’t accumulate. The combinations lead, 24 hpi, to either a rapid decrease in bacterial titers (-), a weak and transient increase (+) or a strong increase in bacterial titer (+++). **(B)** Model of *A. thaliana* non-host resistance against *E. amylovora*. The T3E DspA/E and other elements trigger apoplastic ROS and other defenses that can be repressed by co-inoculation with CHX. CHX: cycloheximide, Dsp: DspA/E, *Ea*: *E. amylovora*, ap: apoplast, cy: cytoplasm, RPL: ribosomal protein, dashed lines: defense that reduces bacterial titers, continuous lines: defense repression that increases bacterial titers. Numbers in between brackets indicate the reference of the corresponding work.

We have previously shown that CHX, an inhibitor of eukaryotic protein synthesis, strongly increases *E. amylovora* bacterial titers in *A. thaliana*. Co-inoculation with CHX suppressed callose deposition (Moreau et al., 2012), a cell-wall based defense normally suppressed during disease in host plants (DebRoy et al., 2004). However, the callose-deficient *pmr4* mutant did not allow an increase in bacterial multiplication, indicating that suppression of callose deposition was, at least not exclusively, the means by which CHX allowed *E. amylovora* multiplication (Moreau et al., 2012). Here we show that co-inoculation of *E. amylovora* with CHX did not suppress intracellular ROS accumulation (**Figure 6A**) but strongly reduced apoplastic ROS accumulation (**Figure 6B**). These data suggest that apoplastic ROS is important for non-host resistance and that intracellular ROS is not. Thus, apoplastic ROS and callose deposition could constitute additive layers of non-host resistance, efficient against *E. amylovora* and both suppressed by CHX (**Figure 7B**).

DspA/E is the major T3E of *E. amylovora* and, in host plants, is considered to be the main trigger of ROS production (Venisse et al., 2003). However, these conclusions are based on the indirect observation that DspA/E leads to an increase in ROS detoxification activity. Our results show that, in *A. thaliana*, a *dspA/E*-deficient strain is able to induce intracellular ROS accumulation but not apoplastic ROS (**Figure 7A**). These data suggest either that DspA/E is not involved in intracellular ROS triggering in the non-host context or that it plays a redundant role with another effector as suggested from data obtained on host plants (Venisse et al., 2003). Concerning apoplastic ROS, our data clearly indicate a role for DspA/E in the triggering of its accumulation in the non-host context. Thus our data demonstrate a specific role for the T3E DspA/E in the triggering of apoplastic ROS. Apoplastic peroxidases are known to play a role in ROS production in response to pathogens (Bindschedler et al., 2006). One could hypothesize that DspA/E increases apoplastic peroxidase activity either trough direct targeting or following an induction of their expression at the transcriptomic level. Indeed, our previous transcriptome analysis (Degrave et al., 2013) showed that expression of DspA/E *in planta* led to an increased expression of *PRX52*, a peroxidase that accumulates in the apoplast in *A. thaliana* leaves infected with the fungus *Verticillium longisporum* (Floerl et al., 2012). Furthermore, peroxidases have been shown to be involved in resistance, which is consistent with our observations that apoplastic ROS is correlated with non-host resistance to *E. amylovora*. For example, *prx33* KO mutants show a clear increase in susceptibility to *P. syringae* (Daudi et al., 2012). Further investigation is required to determine whether apoplastic peroxidases are indeed involved in DspA/E-dependent ROS accumulation and non-host resistance to *E. amylovora*.

Although, DspA/E triggers non-host resistance-associated apoplastic ROS detected by CeCl_3_, our previous data show that DspA/E contributes to the weak bacterial multiplication observed in the non-host context (Degrave et al., 2013; **Figure 7A**). This is consistent with previous work on *N. benthamiana* that indicated a dual role for DspA/E in the non-host context (Oh et al., 2007). This suggests either that in the non-host context, DspA/E is required to induce a susceptibility factor or to repress other defenses, albeit inefficiently. Interestingly, we showed previously that DspA/E, which localizes in the nucleolus, represses protein synthesis and represses the expression of ribosomal-protein (RPL) genes (Degrave et al., 2013). In particular, we have found that expression of *RPL19* was repressed in the 13-1-2 line when DspA/E expression was induced by estradiol (data not shown). Considering the recent results presented by Nagaraj et al. (2015), one could hypothesize that DspA/E targets RPL19 to suppress protein synthesis and thus defense, but that this process is inefficient in non-host plants (**Figure 7B**).

Altogether, our results show that *E. amylovora* triggers both intracellular and apoplastic ROS accumulation (**Figure 7A**). However, consistent with the key role of the apoplast in plant defense (Gupta et al., 2015), only apoplastic ROS accumulation was found to be correlated with non-host resistance. Non-host resistance is generally thought to be a multilayer process that leads to durable resistance because it is more difficult for a pathogen to break than gene-for-gene resistance (Senthil-Kumar and Mysore, 2013). Thus, identifying the molecular actors and understanding the mechanisms of non-host resistance will contribute to the definition of new strategies to develop broad-spectrum durable resistance.

## Author Contributions

AL and MF wrote the manuscript; AL, OP, EW, and MF performed the experiments.

## Conflict of Interest Statement

The authors declare that the research was conducted in the absence of any commercial or financial relationships that could be construed as a potential conflict of interest.
